# Seafood Safety and Quality: The Consumer’s Role

**DOI:** 10.3390/foods5040071

**Published:** 2016-10-28

**Authors:** Doris T. Hicks

**Affiliations:** University of Delaware, Sea Grant College Program, Lewes, DE 19958-1298, USA; dhicks@udel.edu; Tel.: +1-302-645-4297

**Keywords:** seafood, consumers, safe handling

## Abstract

All the good news about seafood—the health and nutritional benefits, the wide varieties and flavors—has had a positive effect on consumption: people are eating more seafood (http://www.seagrant.sunysb.edu/seafood/pdfs/SeafoodSavvy.pdf). Yet consumers want to be assured that seafood is as safe as, or safer to eat than, other foods. When you hear “seafood safety”, think of a safety net designed to protect you, the consumer, from food-borne illness. Every facet of the seafood industry, from harvester to consumer, plays a role in holding up the safety net. The role of state and federal agencies, fishermen, aquaculturists, retailers, processors, restaurants, and scientists is to provide, update, and carry out the necessary handling, processing, and inspection procedures to give consumers the safest seafood possible. The consumer’s responsibility is to follow through with proper handling techniques, from purchase to preparation. It doesn’t matter how many regulations and inspection procedures are set up; the final edge of the safety net is held by the consumer. This article will give you the information you need to educate yourself and be assured that the fish and shellfish you consume are safe. The most common food-borne illnesses are caused by a combination of bacteria naturally present in our environment and food handling errors made in commercial settings, food service institutions, or at home.

## 1. Introduction

As with other perishable foods, foodborne illness caused by microorganisms or naturally occurring toxins is the primary food safety risk associated with seafood. Illness is usually associated with improper harvesting, handling, storage or preparation. Those seafood products that are consumed raw or partially cooked represent the highest risk. Other risks associated with environmental contaminants could be a concern for some individuals especially those who catch and eat their own fish or shellfish from lakes, rivers, streams or bays or harbors that are contaminated by environmental pollutants. The role of state and federal agencies, fishermen, aquaculturists, retailers, processors, restaurants, and scientists is to provide, update, and carry out the necessary handling, processing, and inspection procedures to give consumers the safest seafood possible. The consumer’s job is to follow through with proper handling techniques, from purchase to preparation. It doesn’t matter how many regulations and inspection procedures are set up; the final edge of the safety net is held by the consumer.

A wide variety of seafood products are available in the U.S. marketplace from many different sources. In the U.S., wild fish and shellfish are harvested by commercial fishermen in both near shore and open ocean waters, and in fresh water lakes or rivers. Farm raised (aquacultured) seafood products are raised both on land in ponds (catfish), or re-circulating tanks (tilapia and hybrid bass), and in near shore coastal waters (salmon and shellfish). These same methods are used to farm a wide variety of fish and shellfish in other countries around the world which are then imported into the U.S.

Once seafood products are harvested, they are generally processed or packaged for distribution to retail stores and restaurants. Wild fish and shellfish are unloaded from harvest vessels and farmed products are harvested from facilities then transported and packed for distribution to processing plants or wholesalers. Processors convert the whole fish or shellfish to various other product forms such as fresh fish fillets or steaks or other items such as frozen products, breaded fish portions, and canned or smoked products. Some of these products may be further converted by secondary processors to heat and serve or ready-to-eat products like seafood salads, entrees or other items. Wholesalers and foodservice distributors receive both raw and processed products from many different domestic and foreign sources and distribute them to retail stores and restaurants. Consumers purchase these products from retail stores for home consumption or at restaurants and other foodservice establishments.

The seafood safety and quality guidelines covered in this consumer oriented article are based on messages delivered to consumers time and again over the course of the past 34 years by the author, as the seafood technology specialist with the Delaware Sea Grant Program.

## 2. Seafood Inspection—Industry’s Role

Fish and shellfish, just like milk, baked goods, fruits, vegetables, and groceries of all types, are subject to the Food, Drug, and Cosmetic Act, administered by the U.S. Food and Drug Administration (FDA). The FDA inspects seafood processing plants to ensure compliance with sanitation and food safety regulations, monitors seafood products for conformance to regulations governing pesticides and other contaminants, and maintains extensive surveillance of imported seafood products at their port of entry. In 1997, the FDA Office of Seafood announced a mandatory fish inspection program that is based on the Hazard Analysis Critical Control Point (HACCP) system. Under this system, seafood is monitored at critical points in its journey from sea to consumer to ensure quality and safety. The FDA also updated the Model Food Code, which is designed to help state and local governments prevent food-borne illness. The code incorporates HACCP principles and outlines practices for safe food handling at the retail level. There are several other programs in place to ensure the safety of our seafood. For example, state agencies monitor water quality in shellfish-growing areas to make sure the waters meet the safety standards for the safe harvest of shellfish.

### 2.1. What is HACCP?

HACCP (pronounced “has-sip”) is an acronym for Hazard Analysis Critical Control Point. It’s an effective way of ensuring the safety of food. It works by preventing food safety problems from developing rather than testing food after production to see if it is safe. There are two parts to HACCP. Part one includes making a list of things that can cause the food to be unsafe—we call this hazard analysis. Part two is deciding at which place in the production of the food the hazards can best be controlled—we call this the critical control point for that hazard.

### 2.2. How Does HACCP Make Seafood Safe?

All parts of the seafood processing operation are examined for hazards including raw materials, ingredients, processing steps, storage, and distribution. Hazards include disease causing organisms, toxins, environmental contaminants (such as pesticides), chemicals (cleaners, sanitizers, lubricants, etc.), and physical hazards (wood, metal, and glass). For each hazard, a critical control point is identified where the potential food safety problem is controlled. Records are kept at each critical control point so inspection agencies can be certain the HACCP system is operating to provide safe food. As an extra measure of safety, certain sanitation activities also must be conducted and documented. Under the FDA regulations, all seafood processors are required to operate under the HACCP program. All imported seafood is also covered. Overall, the message to consumers about seafood is good. The vast majority of seafood in the marketplace is safe, and most hazards can be eliminated or prevented by proper handling and thorough cooking. This is where the consumer plays a major role. By learning the proper guidelines for buying, handling, storing, and preparing seafood, you can help ensure that there are no holes in the seafood safety net [1].

## 3. The Consumer’s Role—Buying Seafood

What should you know in order to purchase high-quality seafood? First, it’s important to buy seafood from reputable dealers—those with a known record of safe handling practices—and avoid roadside stands. And since seafood is highly perishable, purchase it last. Make sure the raw juices from seafood do not drip on other foods, especially those that will be eaten without further cooking. (Bacteria in the raw juices can cause cooked foods to spoil, and since these foods are already cooked, there won’t be any chance for the bacteria to be destroyed.) You can avoid cross-contamination in your shopping cart by enclosing individual packages of seafood in plastic bags. Note that the word “fresh” refers to seafood that has not been frozen. Yet “frozen” does not have a bad connotation. Frozen seafood can be superior in quality to fresh seafood, so base your purchase on product quality. (Products labeled “fresh frozen” indicate the seafood was frozen while it was fresh, in many instances within hours of harvest. If fishery products were frozen and thawed for retail sale they should be labeled “previously frozen.”) How can you determine the quality of fresh seafood in the store? First, look at the display. All fresh seafood should be held as near to 32 °F as possible, which is maintained by refrigeration and/or ice.

The U.S. Food and Drug Administration currently recommends that pregnant or breastfeeding women and children under age 12 should eat 2 to 3 servings (8 to 12 ounces) of a variety of different kinds of fish and shellfish each week. There is a large variety of different types of fish and shellfish in the marketplace. The most frequently consumed items, including shrimp, salmon, canned light tuna, flatfish, tilapia, oysters, crab, pollock, catfish, clams, scallops, lobster and basa or swai, all have low mercury levels.

The FDA recommends that pregnant or breastfeeding women and young children should not eat four kinds of fish, Shark, Swordfish, King mackerel and Tilefish from the Gulf of Mexico, because they contain higher levels of mercury. Consumption of canned “white” or albacore tuna and fresh tuna steaks should be limited to 6 ounces per week because they can have slightly more mercury.

If you eat fish caught by family and friends in your local lakes, rivers, and coastal areas you should check for any sportfish consumption advisories issued in your state. If no advice is available, eat up to 6 ounces (one average meal) per week of fish you catch from local waters, but don’t consume any other fish during that week. Advisories are available from local and state health departments and the U.S. Environmental Protection Agency.

**Figure 1:**
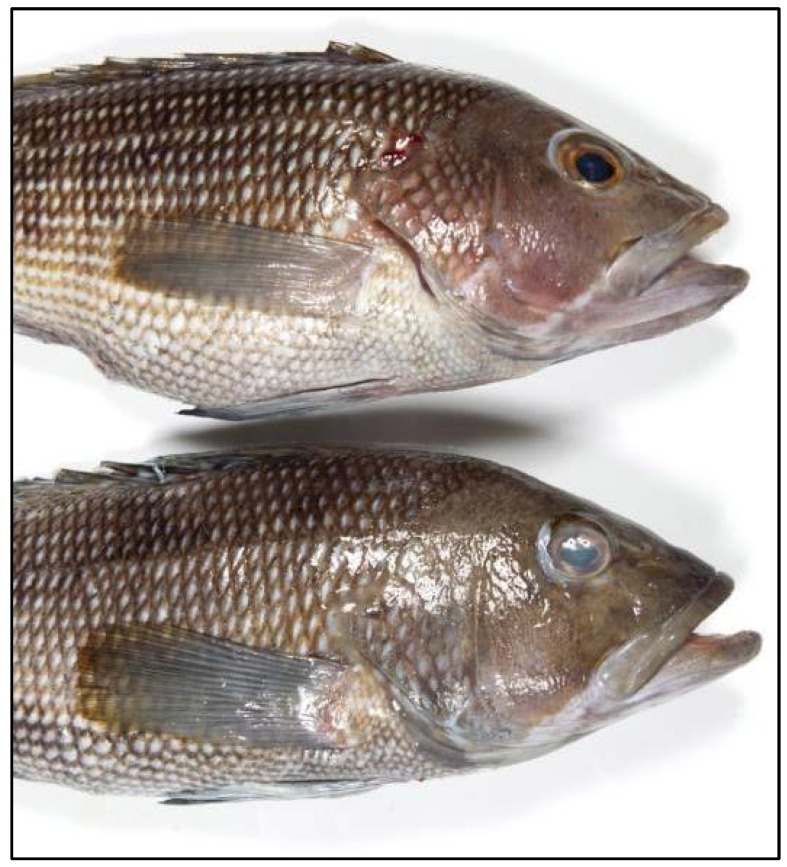
Whole fish: bottom fish is older eyes are clouding over. (Source: Florida Sea Grant).

**Whole Fish.** Whatever the variety, whole fish have certain characteristics that indicate freshness. They should have bright, clear, full eyes that are often protruding. As the fish loses freshness, the eyes become cloudy, pink, and sunken. The gills should be bright red or pink. Avoid fish with dull-colored gills that are gray, brown, or green. Fresh fish should be free of loose or sloughing slime. The flesh should be firm yet elastic, springing back when pressed gently with the finger. With time, the flesh becomes soft and slips away from the bone. The skin of a fresh, whole fish should be shiny with scales that adhere tightly. Characteristic colors and markings start to fade as soon as a fish leaves the water, but the skin should still have a bright, shiny appearance (See Figure 1).

**Figure 2:**
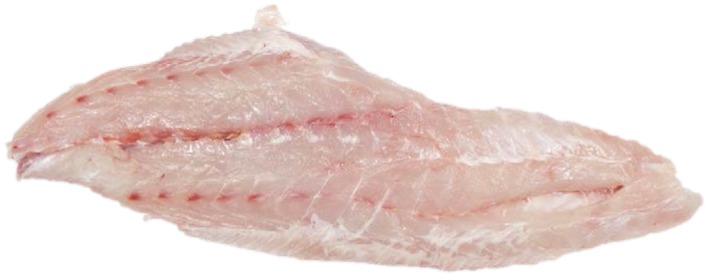
Fresh fish fillet. (Source: Florida Sea Grant).

**Fish Fillets or Steaks.** Note that fillets and steaks should have firm, elastic flesh and a fresh-cut, moist appearance, with no browning around the edges. Fillets separate if they are left too long in the case. The flesh should be almost translucent—as if you can almost see through it. There should be little evidence of bruising or reddening of the flesh from retention of blood. Prepackaged steaks and fillets should contain a minimum of liquid. Fish fillets stored in liquid deteriorate quickly (See Figure 2).

**Shellfish.** They may be sold live, cooked, or fresh-shucked. Each form and species has different quality signs to examine. The shells of live clams, oysters, or mussels should look moist and be tightly closed. If the shells gape slightly, have your retailer tap them. If the shells do not close, do not purchase them. Do not purchase live shellfish with cracked shells. The bottom shell of an oyster should be well cupped—a sign that the oyster inside is plump and well formed. The “neck” or “snout” of soft-shelled clams should show movement. The meats of fresh-shucked clams, oysters, or mussels should be plump and covered with their liquor. Their liquor should be clear or slightly opalescent (slightly milky or light gray) and free of shell or grit. There should be no strong odor.

**Raw Scallop.** Scallops are not usually sold live because they are highly perishable. Typically scallops are shucked at sea shortly after capture. On occasion, day boats will bring whole scallops to market or local restaurants. Fresh scallop meats have a firm texture and a distinctly sweet odor. A sour or iodine smell indicates spoilage. The smaller bay and calico scallops are usually creamy white, although there may be some normal light tan or pink coloration. The larger sea scallops are also generally creamy white, although they may show some normal light orange or pink color.

**Live Crab.** Live crabs and lobsters should show leg movement, and the tail of lobsters should curl tightly underneath the body and not hang down when the lobster is picked up. Lobsters and crabs will not be very active if they have been refrigerated, but they should move at least a little bit.

**Cooked Lobster.** Cooked lobsters or crabs in the shell should be bright red and have no disagreeable odor. Picked lobster meat will be snowy white with red tints, while crabmeat is white with red or brown tints, depending on the species or the section of the body it was picked from. Cooked, picked lobster or crabmeat should have good color and no disagreeable odor.

**Raw Shrimp.** Raw shrimp meat should be firm and have a mild odor. The shells of most varieties are translucent with a grayish green, pinkish tan, or light pink tint. The shells should not have blackened edges or black spots—this is a sign of quality loss. Cooked shrimp meat should be firm and have no disagreeable odor. The color of the meat should be white with red or pink tints. Tiger shrimp have bluish colored shells with black lines between the segments of the shell (these are not black spots).

**Squid.** When buying whole squid, look for eyes that are clear and full. The skin should be untorn and the meat very firm. The skin of fresh squid is cream colored with reddish brown spots. As squid ages, the skin turns pinkish and the flesh will yellow.

**Caviar.** The unopened jar or tin of caviar can be stored in the refrigerator up to two weeks. An opened jar or tin of caviar can be stored in the refrigerator, covered, for no longer than two or three days. Most varieties of caviar should be kept refrigerated in the coldest part of the refrigerator, never frozen, with the exception of Salmon caviar, as it best preserved frozen.

**Label-Dated Seafood.** Buy pasteurized crabmeat and other products only if the “sell by” or “use by” date has not expired. While helpful, these dates are reliable only if the seafood has been kept at the proper temperature during storage and handling.

**Mail-Order Seafood.** Gift seafood is a growing specialty market, mainly for gourmet products. Fresh and frozen seafood are also available to people living far away from the resource. Maine lobsters can be shipped anywhere in the United States. Canned salmon, canned chopped clams, seafood seasonings and marinades, and some smoked products are shelf-stable and require no refrigeration. However, any other fresh or frozen seafood product must arrive as cold as if refrigerated in order to be safe. Before ordering such items, ask how and when the product will be shipped, and whether a cold source will be included to ensure that the product will be received cold. Try to be home when your order arrives, so you can put it right in your refrigerator or freezer. If you aren’t home, give specific instructions about where it should be left. If you receive a package containing live shellfish or fresh or frozen seafood, check the item upon receipt to see if the shellfish are alive, the fresh product is as cold as if refrigerated, and the frozen product is frozen. If it is not, call the mail-order company for a replacement that will arrive cold or request a refund.

**COOL (Country of Origin Labeling) for Seafood.** Under USDA regulations all seafood must be labeled as to the Country it was processed in (i.e., Product of USA.) and whether it is wild or farm-raised. Look for these statements on the product packaging or in the retail refrigerated case [2].

## 4. Consumer Handling and Storing Fresh Seafood

The storage life of seafood depends on how well you take care of it, whether it is a whole fish or a live oyster. When your seafood purchase arrives home, store it in the coldest part of your refrigerator at a temperature as close to 32 °F as possible. Many home refrigerators operate at 40 °F; therefore, fish will lose quality faster.

**Fish.** Fish bruises easily, so lift a whole fish with both hands and avoid holding it by the tail. Pack dressed fish on ice in the refrigerator. Seal fillets or steaks in plastic bags or containers; then cover them with ice in trays or pans. Empty the melt water regularly and add more ice as necessary. Fish that is not prepackaged should be washed under cold, running water and patted dry with an absorbent paper towel. The fish should then be wrapped in moisture-proof paper or plastic wrap, placed in a heavy plastic bag, or stored in an air-tight, rigid container until ready for cooking. The shelf life of fish depends on the variety and its quality at time of purchase. In general, you should use fish quickly—within one to two days.

**Shellfish.** Handling and storage guidelines vary according to the variety of shellfish you purchase.

Store live shellfish in a shallow dish covered with damp towels or moistened paper towels. Never put live shellfish in water or in an air-tight container where they could suffocate and die.

Scrub live oysters, clams, and mussels with a stiff brush such as a vegetable brush just prior to shucking or cooking.

Mussels and clams in the shell (live) should be used within two to three days; oysters in the shell, from seven to 10 days. Some shells may open during storage. If so, tap them. They will close if alive; if not, discard them.

Store shrimp, squid, and shucked shellfish in a leak-proof bag, plastic container, or covered jar. Squid and freshly shucked clams have a shelf life of one to two days. Shrimp and scallops have a shelf life of about two to three days. And freshly shucked oysters have a shelf life of five to seven days.

Live lobsters and crabs should be cooked the same day they are purchased. Store cooked whole lobsters or crabs in rigid air-tight containers and use them within two to three days. Cooked, picked lobster or crabmeat may be stored in a sealed moisture- proof plastic bag or air-tight plastic container for three to four days. Pasteurized crabmeat can be refrigerated for up to six months before opening; use it within three to five days after opening.

**Leftovers.** Taking care of leftovers is a critical food handling step and is often where errors can occur, sometimes resulting in food-borne illness. To prevent a problem at this step, wash hands before handling leftovers and use clean utensils and surfaces. Refrigerate or freeze leftovers in covered, shallow (less than 2 inches deep) containers within two hours after cooking. Leave air space around containers to allow circulation of cold air and to help ensure rapid, even cooling. When preparing seafood for later use, refrigerate or freeze it immediately after cooking in covered, shallow containers. Refrigerators and freezers are designed to compensate for the addition of a few temporarily hot foods without allowing other foods to warm up. Refrigerate leftovers within two hours when the temperature in the food serving area is below 90 °F and within one hour when the temperature of the air is 90 °F or above. Date leftovers so they can be used within a safe time as shown in the seafood storage guide (Table 1). Before serving, cover and reheat leftovers to 160 °F. Soups, sauces, and other “wet” foods should be reheated to a rolling boil. If in doubt, throw it out. Discard outdated, obviously spoiled, or possibly unsafe leftovers in a garbage disposal or in tightly wrapped packages [1].

## 5. Buying Frozen Seafood

Commercially frozen fish is quickly frozen at its peak freshness and the consumer can now find a wide choice of top-quality and wholesome seafood in the freezer case. When properly thawed, frozen fish is comparable to fish that was never frozen. Both exhibit the qualities of freshness described previously. Frozen fish and shellfish should be packaged in a close-fitting, moisture-proof package. Select packages from below the load line of the freezer case. Look for packages that still have their original shape and the wrapping intact with little or no visible ice. Seafood should be frozen solid with no signs of freezer burn, such as discoloration or drying on the surface, and have no objectionable odor. The same guidelines apply for frozen prepared seafood, such as crab cakes, breaded shrimp, or fish sticks. Do not allow the package to defrost during transportation.

## 6. Storing Frozen Fish

After shopping, immediately store commercially wrapped frozen seafood in your freezer. Put it in the coldest part of the freezer, at a temperature as close to −20 °F as possible. As with other frozen foods, avoid prolonged storage by planning your purchases, keeping in mind “first in, first out” Commercially frozen seafood can be stored in the freezer for six to 12 months depending on the type of fish and the amount of fat it contains. Freezing fish at home should be reserved for those times when you end up with more than you can immediately eat, such as after a fishing trip or if someone cancels for dinner. Freezing fish or shellfish in the home or commercial freezer will not improve quality; it only maintains the quality of the food at the time it is frozen.

To freeze seafood at home, start with a high-quality and carefully handled product. Fish should be cleaned first under cold water and then patted dry. Wrap with plastic wrap, excluding as much air as possible. Then overwrap your fish with freezer paper or aluminum foil. There are also specially designed plastic bags for use in the freezer. These may also be used for fish. Carefully seal all packages and label with contents, amount, and date. Place the packages in the coldest part of the freezer where the cold air can circulate around them, freezing them quickly. Shellfish such as shucked clams, oysters, or mussels can be frozen in rigid air-tight plastic containers. Be sure the meats are covered with their liquor and there is a 1/2-inch space between the liquid and the container lid to allow for expansion. Scallops may be frozen in plastic freezer bags. Be sure to exclude air and seal tightly or pack scallops tightly in covered freezer containers. Frozen, shucked shellfish can be stored for three to four months. Most shrimp available in the market has been previously frozen. Be sure shrimp has not been frozen if you plan to freeze it. Refreezing shrimp under non- commercial conditions can significantly affect the flavor and texture, and, in some cases, may make the shrimp unsafe to eat when thawed.

The National Fisheries Institute has developed a seafood storage guide (Table 1) for fresh and frozen products. This guide indicates optimal shelf life for seafood products held under proper refrigeration or freezing conditions. Temperature fluctuations in home refrigerators will affect optimal shelf life, as will opening and closing refrigerators and freezers often. Although these storage times ensure a fresh product for maximum refrigeration storage life at 32 °F, the consumer should plan on using seafood within 36 to 48 h for optimal quality. To determine the approximate storage time for species not listed, ask your retailer which category (lean, fat, shellfish, breaded, or smoked) the seafood falls within and refer to the guide.

## 7. Thawing

It is not always necessary to thaw seafood before cooking, depending on how it will be prepared. If thawing is not necessary, simply double the cooking time. But if your recipe calls for coating, rolling, or stuffing, or if the fish is in a block, you will need to defrost it to facilitate handling. Plan ahead; defrost the fish overnight in the refrigerator. This is the best way to thaw fish to minimize loss of moisture. A one-pound package will defrost within 24 h. Never defrost seafood at room temperature or with hot or warm water. Bacteria on the surface will begin to multiply and cause spoilage. If you forget to take your seafood out of the freezer ahead of time, place it in the sink under cold, running water. A one-pound package will defrost in approximately one hour. You may also use your microwave oven to partially thaw your fish. Use the lowest defrost setting, which is usually 30 percent of normal power levels, and follow the manufacturer’s instructions for time based on amount of fish. (A pound of fillets defrosts in five to six minutes.) The fish should feel cool, pliable, and slightly icy. Be careful not to overheat it and begin the cooking process. Foods defrosted in the microwave oven should be cooked immediately after thawing. When thawing frozen fish that comes in a vacuum-sealed package, remove it from the package, cover, or wrap, and thaw it under refrigeration immediately before use. Do not thaw product while it is still inside the vacuum-sealed package.

## 8. Preparation—Keeping It Clean

Finally, it’s time to prepare your seafood! But before you begin, remind yourself of these important sanitary guidelines developed by the U.S. Department of Agriculture Food Safety and Inspection Service. Be sure the food preparation area and all surfaces and utensils that will touch food are clean. Always wash your hands with soap and warm water for at least 20 s before beginning food preparation, before working with new food or new utensils, after finishing food preparation, before serving food, and after going to the bathroom. Do not let juices from raw finfish, shellfish, meat, or poultry come into contact with other foods. Wash cutting board, utensils, counter, sink, and hands with hot, soapy water immediately after preparing raw seafood, meats, or poultry. Also, use a fingernail brush to clean under nails and cuticles. Keep dishwashing sponges and cloths clean. Use cutting boards that are easy to clean—plastic, acrylic, or rubber composition are good choices. Wooden boards may look pretty, but they should only be used for cutting breads because they are porous and difficult to clean thoroughly. Don’t taste any food of animal origin (meat, poultry, eggs, fish, or shellfish) when it’s raw or during cooking. Serve your cooked seafood on clean plates. Never put it back on the plate that held the raw product.

## 9. Cooking—General Rules

Cook fish and shellfish thoroughly. Fish is cooked when it begins to flake and/or loses its translucent (raw) appearance and turns opaque. Cook fish until it reaches an internal temperature of 140–145 °F for 15 s. Follow processor’s directions when preparing frozen, packaged seafood products such as frozen, breaded fish portions. Seafood is usually baked in a moderate to high oven temperature (425 °F). Do not use recipes that call for cooking without a reliable and continuous heat source. Avoid interrupted cooking—completely cook fish and shellfish at one time. Partial or interrupted cooking often produces conditions that encourage bacterial growth. Cooking Shellfish. Be careful not to overcook shellfish. So often shellfish are in small pieces and can easily be overcooked, becoming tough, dry, and flavorless. Some shellfish, such as canned clams or cooked, picked crabmeat and surimi products (imitation shellfish), are already cooked when purchased. In this case, heat the precooked shellfish or surimi product to the desired temperature without cooking further. Scallops and shrimp turn firm and opaque when cooked. It takes from three—five minutes to boil or steam one pound of medium-sized shrimp and three-four minutes to cook scallops. Shucked shellfish, such as clams, mussels, and oysters, become plump and opaque when cooked. The FDA recommends that shucked oysters be boiled or simmered for at least three minutes, fried in oil for at least three minutes at 375 °F, or baked at 450 °F for at least 10 min. Steam clams, mussels, and oysters in the shell for four-nine minutes from the start of steaming. Use small pots to steam shellfish. If too many shells are cooking at once, it’s possible the centers won’t cook thoroughly. Discard any clams, mussels, or oysters that do not open during cooking. Closed shells indicate they may not have received adequate heating. Boiled lobsters or steamed crabs turn bright red. Allow 10–12 min per pound of lobster, starting to time when the water returns to a boil. Steam crabs 25 min when two to three dozen, depending on size, have been placed in a large crab pot.

## 10. Microwave Cooking

Microwave ovens heat food surfaces rapidly. However, time must be allowed for the heat to penetrate to the center of the food. Take the following steps to ensure that food cooks thoroughly and evenly in the microwave oven. Cover the food to hold in moisture and facilitate even cooking. Glass cookware, glass ceramic cookware, and waxed paper are safe for microwave cooking. Plastic wrap may be used to cover containers, but should not touch the food. Before using other types of containers or wraps, check to be sure that they are approved for use in the microwave oven. Unapproved materials may melt, burn, or contain chemicals that can migrate into food during cooking. When following microwave oven cooking instructions on product labels, remember that ovens vary in power and operating efficiency. If you’re unsure of your oven’s capability, try the following test.

**Time to Boil Test.** From a container of half ice and half water, measure exactly one cup of water (no ice) into a glass measuring cup. Place the cup in the center of the microwave oven. Heat on high for five minutes until the water begins to boil. If the water begins to boil in less than three and a half minutes, consider your oven “high power;” if longer, the oven is “low power.” When using a recipe that states a heating time of six to eight minutes, the “high-power” oven will microwave in the shorter time (or six minutes) while the “low-power” oven will take the longer time (or eight minutes).

If the microwave oven doesn’t have a turntable, turn the entire dish several times during cooking. Be sure to stir recipes such as casseroles or soups. Allow seafood cooked in the microwave oven to stand for the recommended time. This is necessary to complete the cooking process. Check for doneness before serving.

## 11. Serving Seafood

Seafood can be a delicious addition to your daily meal routine and for special occasions such as buffets, picnics, and bag lunches. To ensure the safety of your seafood, follow the sanitary guidelines listed in the Preparation section of this guide.

**Buffets.** When serving for a buffet, serve hot food from chafing dishes or warming trays that maintain the internal temperature of the food at 140 °F or above. For cold foods, nestle the serving dish into a bed of crushed ice. Small platters for replenishing the serving table should be prepared ahead and stored in the refrigerator (at 40 °F or below) or kept warm in the oven (at a setting of 200–225 °F). Discard any foods that have been held at room temperature for more than two hours. Fresh food should not be added to a serving dish or platter containing foods that have already been out for serving.

**Picnics and Lunches.** When going on a picnic or traveling with food, keep all perishables in a cooler with ice or freeze-pack inserts until serving time. Make sure the food is cold or frozen to the touch before placing it in a cooler or cold thermos. When packing a “bag lunch” that will be eaten within several hours, placing ice cubes in a re-sealable bag or a small freeze-pack insert in an insulated bag should be all that is necessary to keep the food cold. Be sure to put the cooler or lunch bag in the coolest place possible. Don’t leave it in the direct sun or in a warm car.

## 12. Extra Care for Special Seafood

Seafood is highly perishable and in many cases requires certain precautions when handling for home use. Some seafood products require extra care either because they are more vulnerable to bacteria that can cause food-borne illness or they have unusual characteristics because of the way they are processed. This section provides additional information on handling some “extra care” products.

**Smoked Seafood.** Most of today’s smoked seafood products are lightly smoked to enhance flavor and not to prolong shelf life. Smoked seafood should be refrigerated at all times and stored no longer than four or five days. In the store, smoked seafood should be displayed in a refrigerator case, but not directly on ice. It should not be in direct contact with fresh seafood. Some other things to look for when buying smoked seafood include a firm, springy texture, glossy surface, smoky odor, no traces of dried blood or viscera, and no traces of salt crystals. For longer storage, smoked seafood can be frozen for two to three months.

**Surimi Seafood.** Surimi is the raw material with which imitation seafood is made. Surimi is prepared under strict controls at sea or onshore. Freshly caught Alaskan pollock is filleted, minced, washed, and strained to yield a concentrated fish paste. Small quantities of salt, sugar, and/or sorbitol are added to stabilize the protein during frozen storage. Next, the surimi is processed into food products by blending it with binders such as starch or egg white. Real shellfish, a shellfish extract, or artificial shellfish flavoring is added to make it taste like shellfish. Then it is formed into the desired shape and texture and cooked. Surimi products should look like the cooked form of the fish and shellfish they are meant to resemble. Since they are fully cooked, add these products to your recipe in the last minutes of cooking, leaving just enough time to heat through. When buying imitation seafood, look for opaque off-white body meat and red, cooked-shellfish color on the surface. If the surimi product is frozen, there shouldn’t be crystals in the package—they indicate freeze-thaw problems. When thawed, these products should be moist and firm, not wet and soft. Do not buy products with off odors (sour, fermented, or sulfur smells). This indicates spoilage. It is wise to read the ingredient statement on the label if you are allergic to any fish or shellfish. Surimi seafood should be stored in the refrigerator for no longer than 14 days (follow the manufacturers “use by date” if present on unopened package), or frozen for 9–12 months. (See the seafood storage guide.) Remember, this product is fully cooked. Use sanitary handling techniques to prevent cross-contamination with raw seafood and meat.

**Value-Added Seafood.** Value-added seafood includes battered and breaded seafood, smoked seafood, dried fish, precooked seafood entrees, fresh minced clams, pre-seasoned fish fillets (such as farm-raised catfish), and others. All these products are semi-prepared and refrigerated or frozen to save you steps when preparing meals at home. Keep in mind the safe handling guidelines, cleanliness, and proper storage and cooking temperatures, and always read the label and follow the manufacturer’s directions, especially as new products are developed and reach the marketplace.

To use refrigerated, prepared seafood safely, when purchasing it, make sure the seafood is cold. Also check the “sell by” or “use by” date on the package. Read the label and follow storage and cooking or heating instructions carefully. Use these products within the recommended length of time. When freezing these products, do so as soon as possible after purchase.

**Marinades.** Follow these guidelines when you use marinades to flavor fish and shellfish. If your recipe calls for basting cooked fish or shellfish with marinade, reserve a portion of it for this before combining the marinade with the raw seafood. Marinate seafood in the refrigerator in a glass or plastic container. Marinades often contain acidic liquids such as wine, lemon juice, or vinegar, which react with metal. Avoid cross-contaminating other foods by thoroughly cleaning any utensils, bowls, or surfaces the marinade comes in contact with after it is combined with raw seafood. Do not save marinades that have been combined with raw seafood, unless they will be immediately cooked in a sauce. Bring the marinade to a rolling boil before adding any other ingredients. Then cook the sauce to at least 160 °F.

## 13. The Final Edge of the Safety Net

The most important considerations in safe handling of seafood at home are cleanliness, temperature, and time. Keep your hands, preparation area, and utensils clean. Avoid cross-contamination. Never let raw seafood come in contact with cooked seafood or other raw or cooked foods. Be aware of temperatures—of the air, of your refrigerator and freezer, of cooking, too. Use the temperature guide in this publication. And be aware of time—the clock starts when fish and shellfish leave the water. Finally, to keep your seafood safe, buy high-quality products and just like they say in the industry, keep it clean, keep it cool, and keep it moving! By following these rules, you can feel confident in holding up your edge of the seafood safety net.

## Figures and Tables

**Table 1:** Seafood Storage Guide [2].

Seafood Storage Guide				Purchased		
				Frozen/Kept Frozen	Fresh/Frozen at Home	Fresh or Thawed/Kept Refrigerated
Product	Fish Fillets/Steaks	Lean	Cod, Flounder Haddock, Halibut	10–12 months	6–8 months	36 h
			Pollock, Ocean Perch	8–9 months	4 months	36 h
			Sea Trout, Rockfish			
			Pacific Ocean Perch			
		Fat	Mullet, Smelt	6–8 months	N/A *	36 h
			Salmon (cleaned)	7–9 months	N/A	36 h
	Shellfish	Dungeness Crab		6 months	6 months	5 days
		Snow Crab		6 months	6 months	5 days
		Blue Crab Meat (fresh)		N/A	4 months	3–5 days
		Blue Crab Meat (pasteurized)		N/A	N/A	6 months
		Cocktail Claws		N/A	4 months	5 days
		King Crab		12 months	9 months	7 days
		Surimi Seafoods		10–12 months	9 months	2 weeks
		Shrimp		9 months	5 months	4 days
		Oysters (shucked)		N/A	N/A	4–7 days
		Clams (shucked)		N/A	N/A	5 days
		Lobster (live)		N/A	N/A	1–2 days
		Lobster (tail meat)		8 months	6 months	4–5 days
		Squid		8-9 months	4 months	36 h
	Breaded Seafood	Shrimp		12 months	8 months	N/A
		Scallops		16 months	10 months	N/A
		Fish Sticks		18 months	N/A	N/A
		Portions		18 months	N/A	N/A
	Smoked Fish	Herring		N/A	2 months	3–4 days
		Salmon, Whitefish		N/A	2 months	5–8 days
	Roe Caviar [3]	Salmon, Fresh Frozen		2 months	2 months	N/A
	Roe Caviar [3]	Salmon, Pastuerized		N/A	2–3 weeks	2–3 days

* Not Applicable or Not Advised.

## Appendix A

Additional reading:

Response to the Questions Posed by the Food and Drug Administration and the National Marine Fisheries Service Regarding Determination of Cooking Parameters for Safe Seafood for Consumers [4].
Food-Borne Illness: A Guide to Safe Food Handling [5].
Delaware Sea Grant [6].
Seafood Health Facts [7].
Salmon Roe Caviar [8].
Fresh and Frozen Seafood: Selecting and Serving It Safely [9].
